# Antidepressant-like effects of the aqueous macerate of the bulb of *Gladiolus dalenii* Van Geel (Iridaceae) in a rat model of epilepsy-associated depression

**DOI:** 10.1186/1472-6882-13-272

**Published:** 2013-10-20

**Authors:** Gwladys Temkou Ngoupaye, Elisabeth Ngo Bum, Willie Mark Uren Daniels

**Affiliations:** 1 Discipline of Human Physiology, School of Laboratory Medicine and Medical Sciences, University of KwaZulu-Natal, Durban, South Africa; 2 Department of Biological Science, University of Ngaoundéré, Ngaoundéré, Cameroon; 3 Department of Animal Biology, University of Dschang, Dschang, Cameroon; 4 Laboratory of Physiology and Phytopharmacology, Department of Animal Biology, University of Dschang, P.O. BOX: 67, Dschang, Cameroon

**Keywords:** Depression, Epilepsy, HPA axis, BDNF, *Gladiolus dalenii*

## Abstract

**Background:**

In Cameroonian traditional medicine various extracts of *Gladiolus dalenii* Van Geel (Iridaceae) have been used as a cure for various ailments that include headaches, digestive problems, muscle and joint aches, and some central nervous system disorders such as epilepsy, schizophrenia and mood disorders. Owning to this background, the aim of the study was to investigate whether an aqueous macerate of the bulb of *Gladiolus dalenii* has any antidepressant activity focusing specifically on depression-like behaviours associated with epilepsy.

**Method:**

We used the combined administration of atropine and pilocarpine to rats as our animal model of epilepsy. The forced swim test and spontaneous locomotor activity in the open field test were the two tools used to assess the presence of depression-like behaviour in epileptic and control animals. The following depression-related parameters were determined: plasma ACTH, plasma corticosterone, adrenal gland weight and hippocampal levels of brain-derived neurotrophic factor (BDNF). The effects of *Gladiolus dalenii* were compared to that of fluoxetine.

**Results:**

Our results showed that we had a valid animal model of epilepsy-induced depression as all 3 measures of construct, predictive and face validity were satisfied. The data indicated that *Gladiolus dalenii* significantly reduced the immobility times in the forced swim test and the locomotor activity as assessed in the open field. A similar pattern was observed when the HPA axis parameters were analysed. *Gladiolus dalenii* significantly reduced the levels of ACTH, corticosterone, but not the adrenal gland weight. *Gladiolus dalenii* significantly increased the level of BDNF in the hippocampus. In all parameters measured the effects of *Gladiolus dalenii* were significantly greater than those of fluoxetine.

**Conclusion:**

The results show that *Gladiolus dalenii* has antidepressant-like properties similar to those of fluoxetine in epilepsy-associated depressive states. The antidepressant activity of *Gladiolus dalenii* is likely to be mediated by restoring the activity of the HPA axis and increasing the levels of BDNF in the hippocampus.

## Background

A recent World Health Organization (WHO) mental health survey showed that mood disorders remain one of the most disabling medical illnesses. The unmet needs of many mental disorders are still a concern and the distribution of resources to mental health in the health fraternity is still suboptimal [1]. Among the mood disorders, depression has always been problematic, especially in terms of overall management and treatment [2]. Despite substantial research into mood disorders, depression associated with epilepsy has received insufficient attention. For instance the pharmacological mechanisms of many antidepressant drugs have been well documented [3]. However there is a notable paucity of data on the effectiveness of antidepressants in epilepsy-associated depression. In addition, an empiric use of antidepressants for the treatment of depression in patients with epilepsy has been criticized as being based on the assumption that the depressive state in epileptics is exactly the same in nature as depression in non-epileptic patients [4]. Furthermore, commonly used antidepressant therapy is effective in 60–70% of patients and produces a variety of unwanted side effects [3]. It is therefore evident that more research is required to address some of these concerns with respect to depression associated with epilepsy.

Despite the availability of different approaches for the discovery of therapeuticals, natural plant products still remain one of the best sources of new structural types [5]. For instance the “omics” technology: genomics, proteomics, transcriptomics, and lately metabolomics, have made it possible to validate the use of traditional medicines scientifically, identify new bioactive compounds, elucidate their mechanisms of action, and assess toxicity. Moreover the World Health Organization has estimated that in developing countries, medicinal plants contribute significantly to primary health [6]. Indeed the number of people seeking alternative therapies is growing, partially because of the fear of unwanted side effects [7]. Plant-based extracts have therefore become sought after sources of bioactive compounds despite a lack of scientifically based evidence of their healing benefits. However the continued used of medicinal herbs as treatment modalities for pathological conditions demands that these therapeutic claims be scientifically investigated [7].

*Gladiolus dalenii* Van Geel belongs to the family of Iridaceae and is called “Mantsap Letoupuh” (wild onion) in Babadjou language (local language in the western region of Cameroon). It is an ornamental erect robust herb with sword-like leaves and inflorescences to over 1 m high from a woody corm. The corm, 2.5-3.5 cm in diameter, with coriaceous tunics, fragments irregularly. It is straw-coloured and has an outer layer that sometimes becomes fibrous [8]. *G. dalenii* occurs virtually throughout the grasslands, savannas and woodlands of sub-Saharan and southern Africa [9]. It is used to treat a wide range of conditions including, headache, epilepsy, convulsions, intestinal spasms, as an antidote for venomous stings and bites, arthritis, rheumatism, naso-pharyngeal affections and as a laxative [9,10]. In addition, a decoction of the corm of *G. dalenii* has been suggested as a cure for dysentery and dysmenorrhoea [9]. The bulbs of the plant are traditionally used for the treatment of epilepsy and schizophrenia in Cameroon. This function of *G. dalenii* has recently been confirmed by Ngoupaye et al. [11].

The aim of our study was therefore to investigate whether extracts of *G. dalenii* may have any antidepressant properties. We compared its effect to fluoxetine, a selective serotonin reuptake inhibitor (SSRI) that is frequently used to treat depression [12]. We specifically focused on epilepsy-associated depression due to its high prevalence in sub-Saharan-Africa. Indeed temporal lobe epilepsy (TLE) is one of the most common forms of intractable epilepsy. Administration of pilocarpine to rodents results in generalized convulsive status epilepticus (SE) and represents the characteristic neuropathology of patients with TLE [13,14]. Similar to humans, adult rats treated with pilocarpine exhibit spontaneous recurrent seizures during the remainder of their life [15]. This rodent model appears to be highly isomorphic with the human disease, and has subsequently been used in many laboratories since its first description a quarter of a century ago [16-18].

In the present study we investigated the effects of an aqueous macerate of the bulb of *G. dalenii* on depression-like behaviors in an atropine-pilocarpine rat model of epilepsy. We evaluated the integrity of the hypothalamic-pituitary-adrenal (HPA) axis in these animals by measuring the levels of plasma ACTH, plasma corticosterone, and adrenal gland weight. Finally BDNF levels in the hippocampus were assessed, as this brain area is important in the regulation of mood and emotion [19], and increases in BDNF levels were proposed as a mechanism of action for some antidepressant drugs [20].

## Methods

### Plant collection, identification and extract preparation

The corms of *Gladiolus dalenii* Van Geel used in this study were harvested during the dry season from Babadjou (West Cameroon). The botanical identification was performed at the botanical herbarium of Yaoundé. Voucher specimen N° 25742/SRF/Cam has been deposited at the Yaoundé Herbarium. The corms were selected and crushed at room temperature. The paste (100 g) was macerated in 100 ml of distilled water for 5 h. The supernatant (macerate) was then collected and filtered using Whatman N° 1 filter paper. After filtration, the macerate was placed in a dry oven at 35°C and 15 g of a brown solid extract was obtained. The yield of the extraction was therefore 15%.

### Animals

The experiments were performed in the Biomedical Resource Unit of the University of KwaZulu-Natal, Durban, South Africa. All experimental procedures were approved by the Ethics Committee for Laboratory Animals (Ethics approval number 090/11/ Animal). A total number of 45 male Wistar rats weighing 250-300 g were used for the experiments. Rats were housed under standard laboratory conditions (12 h/12 h light/dark cycle with lights on at 06 h00; room temperature of 22 ± 2°C and relative humidity of 70%). Food and water was available *ad libitum*.

### Drugs and treatments

The following drugs were used in the study: pilocarpine (350 mg/kg, Sigma-Aldrich, St. Louis, USA) [15], atropine (1 mg/kg, Sigma-Aldrich, USA) [15], and fluoxetine (15 mg/kg, Eli Lilly, South Africa). Pilocarpine and atropine were given by intraperitoneal route while saline, fluoxetine and *G. dalenii* were given by gavage. Animals that displayed seizure activity were given an intraperitoneal injection of diazepam (Roche Products, France) at a dose of 4 mg/kg [15]. A total of 25 animals were used in our experiments, i.e. 5 groups of animals for each of the 5 different treatments. Group 1 was only handled, Group 2 received atropine and pilocarpine with no further treatment, Group 3 was given atropine and pilocarpine, as well as saline for 7 days, Group 4 received atropine and pilocarpine followed by fluoxetine (15 mg/kg) for 7 days, and Group 5 was treated with atropine and pilocarpine prior to the administration of *G. dalenii* extract (15 mg/kg) for 7 days. This dose of *G. dalenii* was used as it had the most effective response during the screening procedure (data not shown).

### Induction of temporal lobe epilepsy (TLE)

Epilepsy was induced by injecting 40 rats intraperitoneally (i.p.) with pilocarpine (350 mg/kg). Thirty minutes prior to the pilocarpine injection, the animals were treated with atropine (1 mg/ kg, i.p.) to reduce peripheral effects of pilocarpine. Within 20–40 minutes following the pilocarpine injection, the animals started to develop seizures akin to status epilepticus (SE). The display of seizures was allowed to continue for 1 h before the animals were treated with diazepam (4 mg/kg, i.p.). Immediate behavioural observation was continued for at least 5 h after the pilocarpine injection. Animals were expected to partially recover from this initial treatment with diazepam within 2–3 days. However their behaviour was regularly monitored for the next 3 weeks. At the end of the 21 days after the pilocarpine treatment, the rats were video monitored assisted by an experimented observer continuously for a further 72 h in order to assess the recurrence of seizures. Subsequent seizure activities were rated according to the Racine Scale [21]. In brief, seizures were scored by viewing behavioural postures (i.e. lordosis, straight tail, jumping/running, forelimb clonus and/or rearing) during fast forward assessment of the videos. Animals that exhibited at least 2 recurrent seizures per day were selected for further experimentation [15]. These animals were subsequently observed for seven days to investigate the development of mood changes.

At the end of the experiment 20 rats were selected for further experimentation.

### Forced swim test

The forced swim test is a well-characterized model used to screen the effectiveness of antidepressant drugs in rodents [22]. In short, cylindrical Perspex tanks (50 cm height, 18 cm diameter), filled to a depth of 30 cm with water that was kept at constant temperature of 22 ± 1°C, was used. Testing was performed in two phases, the induction phase and the test phase. During the induction phase, animals were placed in the water for 15 min, towel dried and returned to their home cages. After 24 h, the rats were placed in the same tanks for 5 min. The movements of the rats were recorded and the duration of immobility (sec) was measured during this second test phase by an experienced observer that was blind to the experimental conditions. In order to minimize interference with the animal’s behavior, the observer remained at the same location in the room during all trials [23]. The behavioural variable “immobility” was defined as: making no movements for at least 2 sec or making only those movements that were necessary to keep the nose above water. Rats were allowed to move their forepaws slightly or support themselves by pressing their paws against the wall of the cylinder.

### Exploratory activity in the open-field test (OFT)

The spontaneous locomotor and exploratory activities were assessed using the OFT. The apparatus consisted of a plexiglass square box (100 cm width × 100 cm length × 50 cm height), with lines dividing its floor into 25 smaller squares of equal dimensions (20 cm × 20 cm). Each animal was placed individually at the center of the apparatus and observed for 5 min to record locomotor (number of segments crossed with all four paws) and exploratory activities (number of rearings on the hind limbs).

### Neurochemical assays

#### Assessment of hypothalamic-pituitary-adrenal (HPA) axis activity

After the forced swim test in the behaviour room, rats were taken to a separate room where they were allowed to acclimatise for at least 2 h before being decapitated. Trunk blood was collected in EDTA tubes, for plasma ACTH and corticosterone determinations. After collection, whole blood was centrifuged for 10 min in a refrigerated centrifuge at 4°C, and plasma was stored in liquid nitrogen until analysis. Commercially available enzyme immunoassay kits were used to determine ACTH (EIA-3647, DRG International Inc., USA), and corticosterone (MP Biochemicals, Santa Anna, California, USA) levels. The respective assays were performed according to the instructions of the manufacturers.

#### Determination of BDNF

Following decapitation, the hippocampi were dissected from the brain and stored in liquid nitrogen for the determination of BDNF levels using a commercially available ELISA kit (RayBiotech Inc., USA). Samples were weighed and 500 μl lysis buffer added to each sample. Samples were sonicated for 40 sec and then centrifuged at 4°C for 15 min. The supernatant was used for subsequent analysis according to the instructions manual of the manufacturer. The wet weights of the samples were used in calculating the concentration of BDNF in each hippocampus.

### Statistical analysis

Statistical analysis was done using the software program XLstat. The Anderson-Darling test was used to assess the distribution of the data. When the data showed normal distribution parametric tests were used. For three groups analysis of variance (ANOVA) was used followed by Neuman-Keuls test. Where only two groups were compared, Student’s t-test was used. For data that showed a non-normal distribution the Kruskal-Wallis and Mann–Whitney U tests were used. All data are presented as mean ± SEM per group. When the p value was less than 0.05 the difference between groups was considered statistically significant.

## Results

### Reduction of depression-like behaviour in the forced swim test

**Figure 1:**
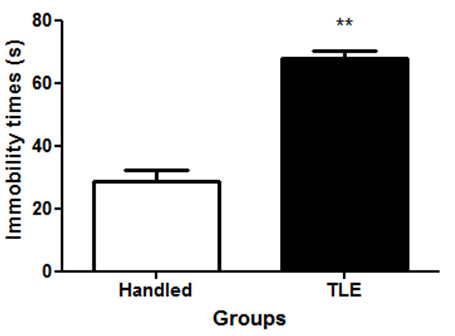
**Immobility times of animals in 5 minutes Porsolt swim test that were just handled and that displayed temporal lobe epilepsy (TLE).** TLE was induced by injecting rats intraperitoneally (i.p.) with pilocarpine (350 mg/kg). Thirty minutes prior to the pilocarpine injection, the animals were treated with atropine (1 mg/ kg, i.p.) to reduce peripheral pilocarpine effects. Results are the mean ± SEM of n = 5 rats per group. ** p < 0.01, significantly different from handling group (Mann Whitney U test).

**Figure 2:**
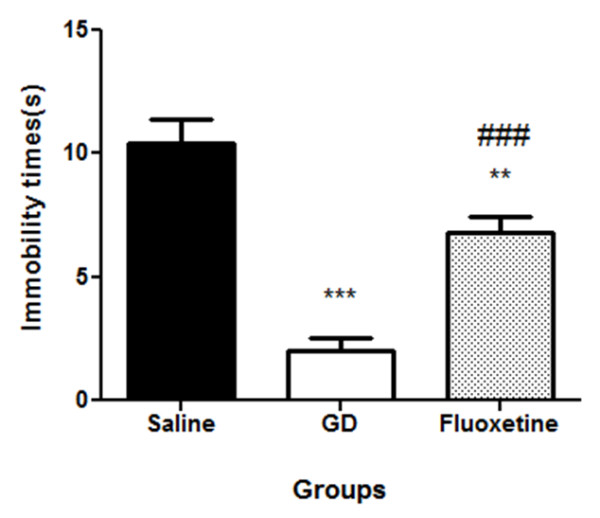
**Immobility times of animals in 5 minutes Porsolt swim test that displayed temporal lobe epilepsy (TLE) and treated with saline, GD and Fluoxetine every day for seven days.** TLE was induced by injecting rats intraperitoneally (i.p.) with pilocarpine (350 mg/kg). Thirty minutes prior to the pilocarpine injection, the animals were treated with atropine (1 mg/ kg, i.p.) to reduce peripheral pilocarpine effects. Results are the mean ± SEM of n = 5 rats per group. ** p < 0.01, significantly different from saline group (ANOVA followed by Neuman-Keuls test). *** p < 0.001 significantly different from saline group (ANOVA followed by Neuman-Keuls test). ### p < 0.001 significantly different from *G. dalenii* treated group (ANOVA followed by Neuman-Keuls test). GD: *G. dalenii*: aqueous macerate of the bulb of *Gladiolus dalenii.*

Animals that received atropine/pilocarpine injections displayed significantly greater immobility times than the control animals that were only handled (Figure 1; p < 0.01). These immobility times were markedly reduced in the animals that were treated either with *G. dalenii* or fluoxetine when compared to saline-treated controls [F (2, 14) = 31.34, p < 0.0001], with *G. dalenii* being more effective than fluoxetine (Figure 2).

### Decrease in locomotor activity in the open field

Animals treated with *G. dalenii* showed a significant decrease in both spontaneous locomotor activity [F (2, 14) = 11.57, p = 0.0016], (number of crossings) as well as exploratory behaviour (number of rearings) [F (2, 14) = 4.680, p = 0.0314] when compared to saline-treated controls, while fluoxetine treated animals displayed levels of spontaneous locomotor activity similar to controls (Table 1).

**Table 1:** **Effect of*G.dalenii*on the locomotor activity assessed on the open field test**

**Treatments**	**Doses (mg/kg)**	**Crossing**	**Rearing**	**Time spent in the center (s)**
Saline	-	30.8 ± 7.86	3.4 ± 1.06	5.6 ± 0.93
GD	15	11 ± 2.33 **	1 ± 0.66 *	6.2 ± 0.86
Fluoxetine	15	33.2 ± 4.46	2.8 ± 0.53	5.2 ± 1.2

Saline, *G. dalenii*, and fluoxetine were administrated every day for seven days. Rats were treated with saline, *G. dalenii* and fluoxetine every day for seven days. The value represent means ± SEM (n = 5 rats per group). *p < 0.05; **p < 0.01 compare to the control group (one way ANOVA followed by Newmann Keuls test).

GD: *G. dalenii*: aqueous macerate of the bulb of *Gladiolus dalenii.*

### Neurochemical assays

#### Assessment of the HPA axis activity

Assessment of the activity of the HPA axis showed that the level of ACTH was significantly increased in the TLE group of non-treated rats when compared to the handled control group (Table 2; p < 0.05). A similar pattern of results was obtained when the plasma concentrations of corticosterone were compared between the two groups of animals (Table 2; p < 0.001). The weights of the adrenal glands of the rats with TLE were significantly elevated when compared to the handled group (Table 2; p < 0.05).

**Table 2:** Activity of the HPA on handled rats and non treated TLE rats

**Groups**	**ACTH (pg/ml)**	**Corticosterone (pg/ml)**	**Adrenal gland weight (Percentage of body weight) (%)**
Handled	0.512 ± 0.114	4126.76 ± 298	0.0076 ± 0.0008
Non treated TLE rats	0.761 ± 0.113*	8267.05 ± 510***	0.0099 ± 0.0014*

TLE was induced by injecting rats intraperitoneally (i.p.) with pilocarpine (350 mg/kg). Thirty minutes prior to the pilocarpine injection, the animals were treated with atropine (1 mg/ kg, i.p.) to reduce peripheral pilocarpine effects. Results are the mean ± SEM of n = 5 rats per group.

* p < 0.05, significantly different from handling group (Student’s t-test).

*** p < 0.001, significantly different from handling group (Student’s t-test).

**Figure 3:**
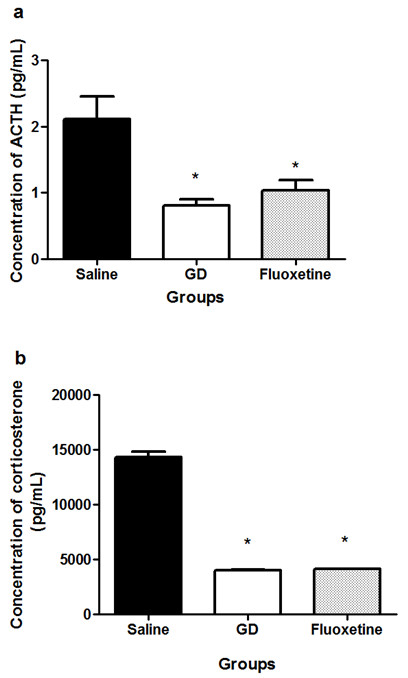
**The plasma concentration of ACTH and corticosterone of rats that were treated with saline, GD and fluoxetine every day for seven days, displayed temporal lobe epilepsy (TLE).** TLE was induced by injecting rats intraperitoneally (i.p.) with pilocarpine (350 mg/kg). Thirty minutes prior to the pilocarpine injection, the animals were treated with atropine (1 mg/ kg, i.p.) to reduce peripheral pilocarpine effects. **a)** The plasma concentration of ACTH; **b)** The plasma concentration of corticosterone. Results are the mean ± SEM of n = 4 rats per group. * p < 0.05, significantly different from saline group respectively (Kruskal-Wallis followed by Mann Whitney U test). GD: *G. dalenii*: aqueous macerate of the bulb of *Gladiolus dalenii.*

**Figure 4:**
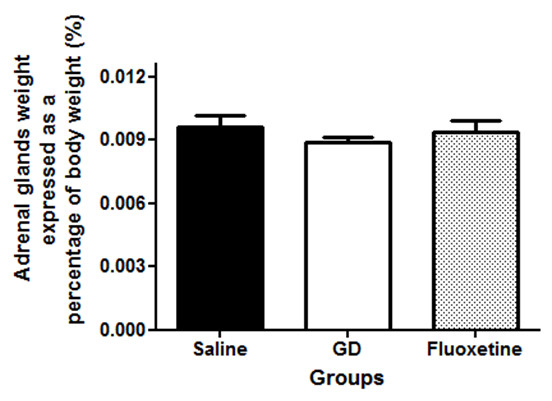
**Adrenal glands weight of animals that displayed temporal lobe epilepsy (TLE) and treated with saline, *G.dalenii *and fluoxetine every day for seven days.** TLE was induced by injecting rats intraperitoneally (i.p.) with pilocarpine (350 mg/kg). Thirty minutes prior to the pilocarpine injection, the animals were treated with atropine (1 mg/ kg, i.p.) to reduce peripheral pilocarpine effects. Results are the mean ± SEM of n = 5 rats per group. GD: *G. dalenii*: aqueous macerate of the bulb of *Gladiolus dalenii.*

Assessment of the activity of the HPA axis of treated rats with TLE showed that the level of ACTH was significantly lower in rats treated with *G. dalenii* or fluoxetine than in rats treated with saline (Figure 3a; p < 0.05). A similar pattern of results was obtained when the plasma concentrations of corticosterone were compared between the various treated groups (Figure 3b). The level of corticosterone was significantly decreased in rats treated with *G. dalenii* (72% reduction) and fluoxetine (71.29% reduction) when compared to saline-treated controls (Figure 3b; p < 0.05). The respective weights of the adrenal glands of rats treated either with *G. dalenii* or fluoxetine were not significantly different as compared to the saline-treated group of animals (Figure 4; [F (2, 14) = 0.9701, p = 0.4069]).

#### Assessment of the level of BDNF

**Figure 5:**
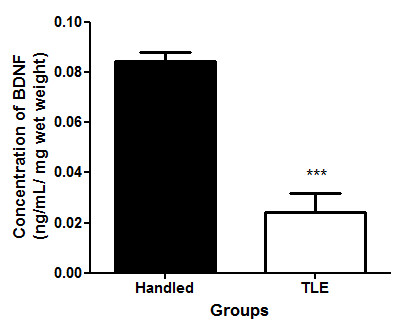
**The concentration of BDNF in the hippocampus of rats that were handled and displayed temporal lobe epilepsy (TLE).** TLE was induced by injecting rats intraperitoneally (i.p.) with pilocarpine (350 mg/kg). Thirty minutes prior to the pilocarpine injection, the animals were treated with atropine (1 mg/ kg, i.p.) to reduce peripheral pilocarpine effects. Results are the mean ± SEM of n = 4 rats per group. *** p < 0.001, Significantly different from Handled group respectively (Student’s t-test).

There were significant changes in the levels of BDNF in the hippocampi of the non-treated rats with TLE. Rats with TLE showed a significant decreased of the level of BDNF when compared to the level of handled rats (Figure 5; p < 0.001).

**Figure 6:**
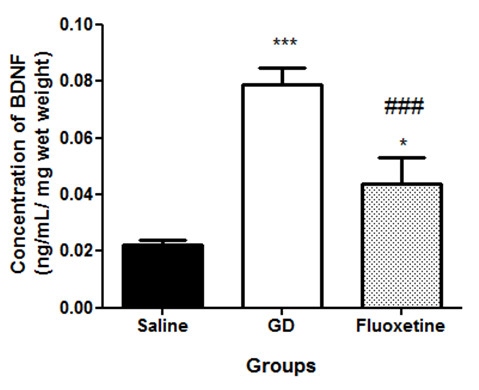
**The concentration of BDNF in the hippocampus of rats that were treated with saline, GD and fluoxetine, displayed temporal lobe epilepsy (TLE).** TLE was induced by injecting rats intraperitoneally (i.p.) with pilocarpine (350 mg/kg). Thirty minutes prior to the pilocarpine injection, the animals were treated with atropine (1 mg/ kg, i.p.) to reduce peripheral pilocarpine effects. Results are the mean ± SEM of n = 4 rats per group. * p < 0.05 and *** p < 0.001, significantly different from saline group respectively (ANOVA followed by Neuman-Keuls test). ### p < 0.001, significantly different from *G. dalenii* treated group respectively (ANOVA followed by Neuman-Keuls test). GD: *G. dalenii*: aqueous macerate of the bulb of *Gladiolus dalenii.*

There were significant changes in the levels of BDNF in the hippocampi of the various treated groups that displayed TLE. Rats treated with *G. dalenii* and fluoxetine showed significantly higher levels of BDNF than saline-treated animals (Figure 6; [F (2, 11) = 126.8, p < 0.0001]), with *G. dalenii* having the greatest effect.

## Discussion

Depression as a common comorbidity of temporal lobe epilepsy (TLE) is poorly understood [24,25]. In general, evidence for treatment strategies of mood disorders in epilepsy are lacking, and development of management approaches tend to rely on clinical experience rather than evidence-based trials favoring one treatment over another [26]. The aim of the present study was therefore to firstly establish an animal model for depression associated with epilepsy, and secondly, to evaluate the potential of an aqueous macerate of the bulb of *G. dalenii* as a possible treatment modality for epilepsy-associated depression.

Our results showed that animals treated with atropine/pilocarpine displayed recurrent seizures. This suggested that the behaviour of these animals were reminiscent of epilepsy in humans and was therefore in agreement with previous studies that reported similar observations [27]. These animals, when subjected to the forced swim test, displayed greater immobilization times when compared to handled controls. An increase in immobility time is usually referred to as behavioral despair in animals, and is claimed to represent human depression [28]. Our animal model therefore showed some face validity.

The immobility times of “depressed, epileptic” animals was significantly reduced when treated with the antidepressant fluoxetine. This result showed that our animal model responded positively to a known treatment and therefore also exhibited predictive validity. Finally, evaluation of some biological parameters revealed aberrations within the hypothalamic-pituitary-adrenal (HPA) axis and hippocampal neurotrophin levels. These neurochemical changes are characterisitic of the depressive state [29] and hence our animal model also showed some construct validity.

Treating our “depressed, epileptic” animals with *G dalenii* reduced their immobility times in the forced swim test to a greater extend than fluoxetine. This finding indicated *G. dalenii* to have antidepressant properties. This notion was further supported by the observation that animals treated with *G. dalenii* showed a decrease in locomotor activity in the open field test similar to previous reports indicating various antidepressants like tricyclic antidepressants and monoamine oxidase inhibitors to reduce locomotor activity of rodents [30,31]. The observed decrease in immobility times could not have resulted from an increase in locomotor activity. Recent studies have confirmed the open field to be a classic animal model to evaluate spontaneous and general activity of animals [32,33]. Our interpretation is therefore in line with other studies attributing reduced immobility times in the forced swim test to antidepressant activity of the drug under investigation [34,35].

In order to confirm our behavioural results, we subsequently assessed the activity of the HPA axis as well as determine the levels of BDNF in the hippocampi of *G. dalenii* treated animals and their respective saline and fluoxetine-treated controls. We found that *G. dalenii* significantly decreased the level of ACTH and corticosterone when compared to the saline treated group. This effect was comparable to that of fluoxetine. Dysregulation of the HPA axis represents an established hallmark of depression [29]. This has been shown by hypersecretion of cortisol and enlargement of the organs including the adrenal glands, due to the trophic effect of ACTH on the adrenal cortex. It is known that excessive activation of the HPA axis is reversed by selective serotonin reuptake inhibitors and other antidepressants [29,36] and therefore the observation that *G. dalenii* administration led to a normalisation of depressive behaviour may be due to its ability to correct the hyperactivity of the HPA axis.

*G. dalenii* administration caused a significant increase in the level of hippocampal BDNF compared to saline and fluoxetine even though a substantial number of preclinical studies have failed to show these changes induced by antidepressants [37,38]. However our finding was in agreement with previous reports demonstrating how chronic administration of several antidepressants, including selective serotonin reuptake inhibitors, increase BDNF expression in the hippocampus [39-41].

It was interesting to note that *G. dalenii* was more effective in decreasing the immobility times and the levels of ACTH, and increasing the levels of BDNF, than fluoxetine. Studies by Mazarati et al. [42] showed that some patients that suffer from epilepsy-induced depression, do not respond well to selective serotonin reuptake inhibitors that included fluoxetine. It may therefore be possible that *G. dalenii* recruits mechanisms additional to serotoninergic pathways to exert its effects. These mechanisms remain speculative and require further investigation.

## Conclusions

The results show that *Gladiolus dalenii* has antidepressant-like properties similar to fluoxetine in epilepsy-associated depressive states. The antidepressant activity of *Gladiolus dalenii* is likely to be mediated by restoring the activity of the HPA axis and increasing levels of BDNF in the hippocampus. Adjunct treatment with *G. dalenii* may therefore be considered for the management of depression associated with epilepsy.

## Abbreviations

ANOVA: Analysis of variance; BDNF: Brain derived neurotrophic factor; GD: Aqueous macerate of the bulb of *Gladiolus dalenii*; HPA: Hypothalamic-pituitary–adrenal; OFT: Open field test; SE: Status epilepticus; SSRI: Selective serotonin inhibitor reuptake; TLE: Temporal lobe epilepsy.

## Competing interests

The authors declare that they have no competing interests.

## Authors’ contributions

GTN is the principal investigator conceived and designed the work, carried out the extract preparation, data collection, analysis and interpretation as part of a requirement for her PhD. ENB assisted in the design of the work. WMUD supervised the work; he has also assisted in the design and the conception of the work. He arranged for the behavioral test and the neurochemical assay, and helped to draft the manuscript. The final version of the manuscript was approved by all authors.

## Pre-publication history

The pre-publication history for this paper can be accessed here:

http://www.biomedcentral.com/1472-6882/13/272/prepub
